# Unique catalytic activities and scaffolding of p21 activated kinase-1 in cardiovascular signaling

**DOI:** 10.3389/fphar.2013.00116

**Published:** 2013-09-27

**Authors:** Yunbo Ke, Ming Lei, Xin Wang, R. John Solaro

**Affiliations:** ^1^Department of Physiology and Biophysics, University of Illinois at ChicagoChicago, IL, USA; ^2^Center for Cardiovascular Research, College of Medicine, University of Illinois at ChicagoChicago, IL, USA; ^3^Faculty of Medicine and Human Sciences, Institute of Cardiovascular Sciences, University of ManchesterManchester, UK; ^4^Faculty of Life Sciences, Manchester Academic Health Sciences Center, University of ManchesterManchester, UK

**Keywords:** scaffolding, auto-phosphorylation, cytoskeletal reorganization, cardiovascular diseases, FTY720

## Abstract

P21 activated kinase-1 (Pak1) has diverse functions in mammalian cells. Although a large number of phosphoproteins have been designated as Pak1 substrates from *in vitro* studies, emerging evidence has indicated that Pak1 may function as a signaling molecule through a unique molecular mechanism – scaffolding. By scaffolding, Pak1 delivers signals through an auto-phosphorylation-induced conformational change without transfer of a phosphate group to its immediate downstream effector(s). Here we review evidence for this regulatory mechanism based on structural and functional studies of Pak1 in different cell types and research models as well as *in vitro* biochemical assays. We also discuss the implications of Pak1 scaffolding in disease-related signaling processes and the potential in cardiovascular drug development.

## INTRODUCTION

In the early 1990s, studies reported by Hall and his colleagues demonstrated significant cytoskeletal reorganization induced by Rho family small GTPases including RhoA, Cdc42 and Rac1 in mammalian cells (Paterson et al., 1990; Hall, 1992; Ridley et al., 1992; Nobes and Hall, 1995). These studies, which revealed dramatic and fascinating cytoskeletal phenotypes, such as formation (or loss) of stress fibers, induction of lamellipodia and filapodia, generated strong enthusiasm to understand the signaling pathways and molecular mechanisms responsible for the cell remodeling. The identification of p21 activated kinase-1 (Pak1) activation by Cdc42 and Rac1 has provided an exciting clue linking the activity of small G proteins to protein phosphorylation, a common post-translational modification regulating virtually all of the major physiological processes. Indeed, an increasing number of phosphoproteins had been claimed as substrates for Paks ever since. Obviously, validation of authentic substrates for Pak requires evidence beyond *in vitro* assays. For instance, Pak1 was found to phosphorylate both myosin regulatory light chain and myosin light chain kinase (MLCK) predictably resulting in both an increase and a decrease of myosin II activity (Sanders et al., 1999; Goeckeler et al., 2000; Zeng et al., 2000), which may not co-exist* in vivo*. And additional complexity in understanding Pak1 function *in vivo *is that mouse models null for Pak1 expression demonstrate phenotypes similar to wild-type mice unless subjected to stresses (Liu et al., 2011; Taglieri et al., 2011).

p21 activated kinase-1 was discovered in rat brain by protein overlay assay as a Cdc42/Rac1 binding partner (Manser et al., 1994). Pak1 and Cdc42/Rac1 induced the same cytoskeletal and morphological changes in mammalian cells (Kozma et al., 1995; Manser et al., 1997; Sells et al., 1997) supporting the hypotheses that Pak1 conveys messages from the small GTPases in signaling cascades. It is evident that understanding Pak1 function *in vivo* requires determination of the native substrates, which may serve as immediate downstream effectors for Pak. Unexpectedly, the search for “physiological” substrates of Pak has been formidable.

It is well accepted that a protein kinase functions through transfer of phosphate group(s) to its downstream target(s). This has been seen in numerous protein kinases as with MLCK, whose name and function is primarily defined by its obligatory protein substrate – myosin regulatory light chain (MLC_20_). Likewise it would seem important with regard to understanding Pak1 control mechanisms, to know its physiological substrate(s). Yet studies with Pak1 mutants cast doubt on the role of phosphorylation of downstream targets as a prominent Pak1 signaling mechanism. These studies demonstrated that kinase dead mutants of Pak1 retained the ability to enhance formation of lamellipodia (Frost et al., 1998) and neurite outgrowth (Daniels et al., 1998; Obermeier et al., 1998). The phenotypes were expected to be stimulated by Pak1 activation as active Cdc42/Rac1 and Pak1 induce the same morphological changes. Frost et al. (1998) were the first to use the term “scaffolding” to describe the kinase-independent activities of Pak1 regulation of cytoskeletal reorganization. Scaffolding may also be induced by auto-phosphorylation of Pak1 as discussed below. In signal transduction, it is a commonly-used scheme of activation as seen in β-arrestin-mediated signaling (Pierce et al., 2001; Tohgo et al., 2002) and in Ca^2+^ triggered signal transduction from troponins to tropomyosin (Parmacek and Solaro, 2004). Is it possible that a protein kinase carries out signal transduction without phosphorylating its downstream target? To address this question, we discuss Pak1 structure and its mode of activation.

## STRUCTURE AND ACTIVATION MECHANISM

**FIGURE 1:**
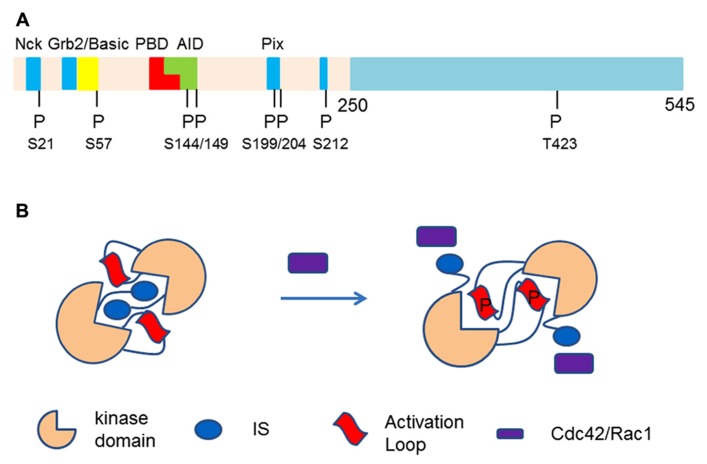
**(A)** p21 activated kinase-1 is divided into N-termial and C-terminal halves. The N-terminal half contains p21 binding domain (PBD) followed by a kinase inhibitory domain which overlap with each other. The proline rich motifs interact with different cellular proteins including Nck, Grb2, and Pix, etc. Most of the motifs contain or are followed by auto-phosphorylation sites. T423 is the only auto-phosphosphorylation site in the catalytic domain which lies in the activation loop. **(B)** Activation of Pak1 by Cdc42/Rac1. Pak1 forms a homodimer, an auto-inhibitory configuration. The activation loop is dislocated from the catalytic center of Pak1 by autoinhibitory domain in inhibitory switch domain (IS, inhibitory switch). When Cdc42 or Rac1 binds to PBD domain, the IS is displaced from its inhibitory position and auto-phosphorylation on activation loop occurs.

Pak family serine/threonine protein kinases have six members divided intro group I and group II Paks. Interestingly, although Pak1 and Pak4 belong to two different groups and are less homologous to each other, compared with members in the same groups (Jaffer and Chernoff, 2002), they produce the same cytoskeletal changes in mammalian cells (Sells et al., 1997; Abo et al., 1998). It remains unclear whether Pak1 and Pak4 employ the same molecular strategy to produce the cytoskeletal reorganization. Group I Paks differ from other non-Pak family protein kinases by a combination of three structural features: a motif-rich regulatory N-terminal half, which interacts not only with different cellular partners, but also extensively with the catalytic domain of Paks; multiple auto-phosphorylation sites mostly clustered in the N-terminal half, which contribute to both kinase activation and translocation (Bokoch, 2003); and formation of an antiparallel homodimer, which integrates the first two traits and provides the structural basis for both auto-inhibition and activation (Lei et al., 2000; Bokoch, 2003; **Figure 1**).

Upon activation by upstream signals, accelerated auto-phosphorylation of Pak1 occurs at multiple sites (Manser et al., 1997; Zhao et al., 1998). The catalytic domain of Pak1 shares more than 92% homology with Pak2 and Pak3 (Jaffer and Chernoff, 2002). Dimerization places the activation loop from one Pak1 monomer in the vicinity of the catalytic cleft of the other monomer. In an auto-inhibitory mode, a segment in the auto-inhibitory domain (AID) displaces the activation loop away from key amino acids that carry out the kinase reaction and prevent phosphorylation of T423 in the loop. This inhibition is released by Cdc42 and Rac1 when they bind to the p21 binding domain (PBD) that is upstream of and overlaps with the AID (Wang et al., 2011a; **Figure 1B**). Two auto-phosphorylations in the AID also contribute to kinase activation (Chong et al., 2001; Lei et al., 2005). The Pak1 dimer is a structure that restricts accessibility of a protein substrate to the catalytic cleft (Lei et al., 2000, 2005). Although it is generally accepted that Pak homodimer breaks and separates when stimulated by Cdc42 and Rac1 (Parrini et al., 2002), NMR studies on Pak2 appear not to support this conclusion. In contrast, phosphorylation of Pak2 in the kinase domain promotes dimer formation in solution (Pirruccello et al., 2006).

Currently, most evidence indicates that Pak1 auto-phosphorylation occurs through an inter-molecular mechanism consisting of auto-phosphorylation between two Pak1 monomers (King et al., 2000; Parrini et al., 2002). Therefore, Pak1 must have intrinsic capacity to maintain a dynamic dimer structure well beyond the time that the first auto-phosphorylation occurs as there are at least seven auto-phosphorylation sites. *In vivo*, a protein substrate has to compete with the “uncharged” auto-phosphorylation sites for the catalytic center. In addition, available auto-phosphorylation sites can be replenished by phosphatases (Koh et al., 2002). The following questions remain unanswered: is auto-phosphorylation essential for kinase activities towards an exogenous protein substrate? Is there a site that becomes auto-phosphorylated earlier than any other sites? Is auto-phosphorylation sequential or random or regulated by Pak1 partners? Do different combinations of auto-phosphorylations lead to different modes of activation or functional effects? Are there mutations in Pak1 that can bring about the same conformational changes as well as the same effects on downstream effectors induced by auto-phosphorylation?

Since its discovery in 1994, many phosphoproteins have been assigned as Pak substrates (Bokoch, 2003). The identified proteins regulate a wide variety of cellular processes including proliferation (King et al., 1998; Beeser et al., 2005; Jin et al., 2005), apoptosis (Schurmann et al., 2000), generation of reactive oxygen species (Knaus et al., 1995), contractility and cell remodeling (Edwards et al., 1999; Sanders et al., 1999), etc. Obviously, caution must be taken to extend the results from *in vitro *conditions to the *in vivo* state. In addition, the use of GST-fusion proteins (GST-Pak1) may interfere with auto-phosphorylation, dimerization and substrate specificity of Pak1. These issues emphasize the importance of employing different approaches with the objective to understand Pak1 catalytic activity in mammalian cells and animal models.

## REGULATION OF CYTOSKELETAL RE-ORGANIZATION AND ENDOTHELIAL BARRIER FUNCTION

Induction of cytoskeletal re-organization by Pak1 has been demonstrated by microinjection of plasmids coding different Pak1 mutants in mammalian cells. Pak1 was made constitutively active by the pseudo-phosphorylation mutant, T423E, in the activation loop, by mutations in dimerization domains, by single amino acid change in PBD and by truncation of Pak1 N-terminal region including the AID. The gain of function mutations induce loss of stress fibers and enhanced formation of lamellipodia and filapodia in mammalian cells (Manser et al., 1997; Sells et al., 1997; Frost et al., 1998). How does Pak1 activation induce collapse of F-actin (filamentous actin)? The answer to this question may reside in proteins that regulate actomyosin polymerization and assembly. Phosphorylation of MLC_20_ enhances the activity of myosin II and likely assembly of actomyosin in mammalian cells (Smith et al., 1983; Kamm and Stull, 2001). A more pertinent protein that regulates F-actin dynamics is actin depolymerization factor, ADF/cofilin (Bamburg, 1999). Phosphorylation of ADF/cofilin at Ser 3 prevents its binding to a complex at the barbed end of F-actin and releases its inhibitory effect on formation or prolongation of F-actin. ADF/cofilin also has capacity to sever pre-existing F-actin (Bamburg et al., 1999). The question arises: How does Pak1 affect phosphorylation and activities of MLC_20_ and ADF/cofilin?

The advantage of studying protein phosphorylation using recombinant viruses lies in their property of transfecting and expressing a recombinant protein in nearly 100% of cultured mammalian cells while the viral gene expression is repressed (Kozarsky and Wilson, 1993; Ke et al., 2004). Therefore, change in protein phosphorylation caused by an enhanced or reduced upstream signal can be better detected. Employing a recombinant Semliki Forest virus that expressed constitutively active Pak1, it was observed that phosphorylation of MLC_20_ in BHK-21 cells was reduced (Sanders et al., 1999).

Recombinant adenovirus is an excellent example of nanotechnology being used to study signal molecules in different disease models. Recombinant adenoviruses have a diameter of 70–90 nm. They transfect most of mammalian cells at high efficiency both *in vitro* and *in vivo* and expressed exogenous proteins ranged from a few kD to 200 kD (Kennedy and Parks, 2009). The viral genome exists in mammalian cells in an episomal state without risk of causing insertional mutagenesis. The purified recombinant adenoviruses can be stored over years at -80°C and theoretically, 1 ml of viral preparation has the capacity to infect as many as 10^9^ mammalian cells at a multiplicity of infection (MOI) of 10 and above (Kozarsky and Wilson, 1993). In addition, the tagged recombinant proteins can be monitored easily *in situ *and purified from different types of mammalian cells, sometimes along with their binding partners, by affinity chromatography (Ke et al., 2004). Elucidation of mechanisms in the study of protein kinase A (PKA) and Pak1 activities in endothelial cell barrier dysfunction and cell signaling in cardiomyocytes represents a successful application of recombinant adenoviruses (Ke et al., 2004, 2007b; Lum et al., 1999).

Endothelial monolayer forms a restrictive barrier that regulates communication between blood and tissues. The barrier dysfunction occurs during many pathological conditions including lung injury, ischemic heart diseases, chronic kidney diseases and inflammation. Signaling molecules that affect endothelial barrier function primarily work through one or more of the three major regulatory mechanisms: tight/gap junctions, focal adhesions and internal contractility (Lum and Malik, 1994). All of these can be measured with an instrument called electric cell-substrate impedance sensor (ECIS), which monitors trans-endothelial electrical resistance that dramatically decreases when endothelial cells are challenged by inflammation mediators such as histamine and thrombin (Tiruppathi et al., 1992). The dynamic curve produced by ECIS is shaped and modified by alteration of cell–cell connection, cell–matrix interaction and actomyosin activities. Protein phosphorylation plays an important role in control of barrier function. For instance, multiple lines of investigation have demonstrated that inhibition of MLCK and reduction in MLC_20_ phosphorylation protect endothelial barrier integrity (Dudek and Garcia, 2001). Therefore, it is interesting to know how Pak1 regulates endothelial barrier dysfunction.

**FIGURE 2:**
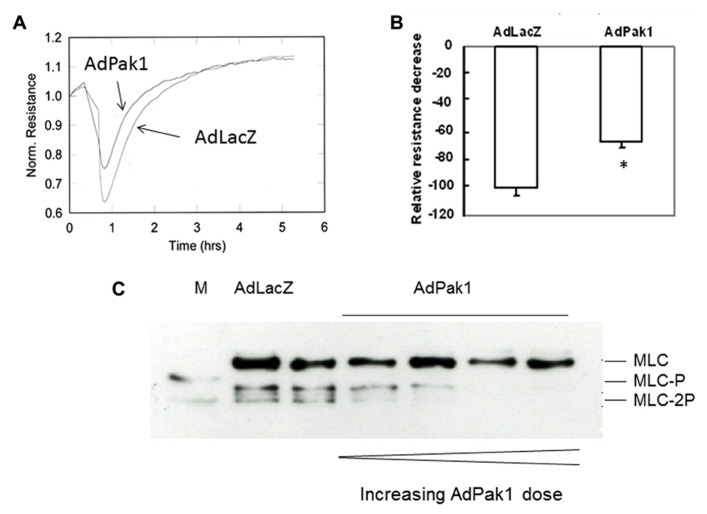
**Endothelial barrier dysfunction is inhibited by expression of constitutively active Pak1 mediated by recombinant adenovirus (AdPak1) through dephosphorylation of MLC_20_.** **(A)** Pak1 reduces thrombin mediated decrease of trans-endothelial electrical resistance as measured by ECIS; **(B)** Bar graph representation of results in A indicates that resistance drop induced by thrombin is rescued by Pak1; **(C)** Pak1 reduces phosphorylation of myosin regulatory light chain (MLC_20_) at both threonine 18 and serine19 through activation of PP2A. M, phosphorylated MLC_20_ as marker; Ctrl, control; AdPak1, recombinant adenovirus expressing constitutively active Pak1.

In endothelial cells, expression of the active Pak1 via recombinant adenovirus induces dephosphorylation of MLC_20_ and inhibits thrombin stimulated barrier dysfunction (Ke et al., 2007b; **Figure 2**). These observations appear to shed light on molecular mechanisms responsible for Pak1 initiated cytoskeletal reorganization inasmuch as phosphorylation of MLC_20_ may regulate assembly of myosin II as well as myosin II activities (Beeser et al., 2005). It was first concluded that Pak1 induces dephosphorylation of MLC_20_ through phosphorylation and inhibition of MLCK because GST-Pak1 phosphorylated MLCK purified from smooth muscle cells and inhibited its activities *in vitro *(Sanders et al., 1999). However, further studies with constitutively active Pak1(caPak1) without the GST tag failed to phosphorylate MLCK of the same source *in* *vitro*. In addition, the caPak1 did not reduce phosphorylation of MLC_20_ in smooth muscle cells (Ke et al., 2007b). In smooth muscle cells, Pak1 induces formation of the podosome, a structure similar to filapodia (Webb et al., 2005). Therefore, dephosphorylation of MLC_20_ may not account for Pak1 induced cell remodeling.

Neuronal growth cone remodeling is regulated by Rho family small G proteins and their downstream effectors. Cdc42/Rac1 and Rho A antagonize each other in regulation of the dynamics of neurite outgrowth (Jalink et al., 1994; Kozma et al., 1997; Brown et al., 2000). The opposite activities are recapitulated by Pak1 and Rock (Daniels et al., 1998; Hirose et al., 1998; Tudor et al., 2005; Boda et al., 2006). In mouse model with ablation of both Pak1 and Pak3 expression, there is an impaired postnatal brain growth associated with reduction in dendrites and axons (Huang et al., 2011). Pak1 and Pak3 may compensate with each other in guiding neuronal morphogenesis (Boda et al., 2008). ADF/cofilin is involved in neurite outgrowth, regulated by Lim kinase and several protein phosphatases, PP1, PP2A and PP2B (Meberg et al., 1998; Ambach et al., 2000; Samstag and Nebl, 2003). Phosphorylation of ADF/cofilin by Lim kinase at serine 3 inactivates its F-actin depolymerization activity accompanied by collapse of the growth cone (Aizawa et al., 2001). Inhibition of growth cone formation was also observed when the phosphatase inhibitor okadaic acid was applied in PC12 cells (Chiou and Westhead, 1992). Studies with ADF/cofilin mutants indicate that ADF activity is critical for promoting neurite extension. While the non-phosphorylatable form of ADF/cofilin induces neurite outgrowth, the pseudo-phosphorylated mutant does not promote the structure (Meberg and Bamburg, 2000), which suggests that dephosphorylation of ADF/cofilin is essential for neurite outgrowth (Meberg et al., 1998). Inhibition of PP1 by Rho A/Rock, which leads to inactivation of ADF/cofilin, is responsible for collapse of the same structure (Hirose et al., 1998; Bito et al., 2000; Lingor et al., 2007). Increased ADF/cofilin activity may initiate accelerated turn-over of F-actin required for establishment of a new structure rich in F-actin (Gallo and Letourneau, 2004; Flynn et al., 2012). Recent studies have demonstrated that Pak1 is important in regulation of t-tubular networks in ventricular myocytes (Desantiago et al., 2013). T-tubules are deep invaginations of the sarcolemma, bringing the extracellular space to the interior regions of the cell for insuring efficient excitation-contraction coupling. It is of interest to know whether there is a common mechanism regulating plasma membrane protrusion and invagination involving Pak1.

## ACTIVATION OF PP2A BY Pak1 THROUGH SCAFFOLDING

An important aspect of Pak1 signaling is its interactions with PP2A. PP2A is a multi-faceted, multi-task phosphatase responsible for dephosphorylation of many different phosphoproteins in the heart and in other mammalian cells (Janssens and Goris, 2001; Ke et al., 2008). It consists of a catalytic subunit (C) with two isoforms, a scaffolding subunit (A) also with two isoforms and a regulatory subunit (B) with at least 18 isoforms. Thus a large number of variations can be formed by different combination of three subunits that associates with distinct protein substrates and carrying out phosphatase activity on these targets regulated in both temporal and spatial manner. At the C terminus of the C subunit, post-translational modifications including methylation at L309 and tyrosine phosphorylation at Y307 regulate PP2A translocation, substrate specificity and phosphatase activity (Janssens et al., 2008). Inhibition of PP2A by okadaic acid induces tumorigenesis (Haystead et al., 1989) and increase pacemaker activity in SA (sino-atrial) node (Kondo et al., 1990).

Association of Pak1 with PP2A was first demonstrated in rat brain. Cross-linking of PP2Ac (the catalytic subunit of PP2A) with its partners in brain extract shifts the Pak1 signal to a higher molecular weight position in SDS PAGE (Westphal et al., 1999). Association of Pak1 with PP2A is not limited to brain tissue. In the heart, both endogenous and constitutively active Pak1 are binding partners for PP2A and expression of the constitutively active Pak1 induces PP2Ac dephosphorylation at Y307. In rat ventricular myocytes, Pak1 activates PP2A and reduces phosphorylation on troponin I, myosin binding protein C, connexin 43 and microtubule-associated protein (MAP4; Ke et al., 2004; Cheng et al., 2010, 2012; Ai et al., 2011). In SA nodal cells, active Pak1 and PP2A demonstrate the same altered pattern of localization (Ke et al., 2007a). Activation of PP2A by Pak1 was also demonstrated in endothelial cells and mast cells (Ke et al., 2007b; Staser et al., 2013).

While phosphorylation of ADF/cofilin promotes polymerization of F-actin, MAP4 phosphorylation stimulates disruption of the microtubule network in cardiomyocytes. A Pak1 induced rise of phosphatase activity leads to increased formation of the microtubule network accompanied by dephosphorylation of MAP4, a tau-like protein (Cheng et al., 2010). An increase of polymerized microtubules could be an indication of ventricular hypertrophy (Tsutsui et al., 1993; Tagawa et al., 1996). On the other hand, myocardial infarction (MI) is often associated with the disruption of microtubule network (Iwai et al., 1990).

**FIGURE 3:**
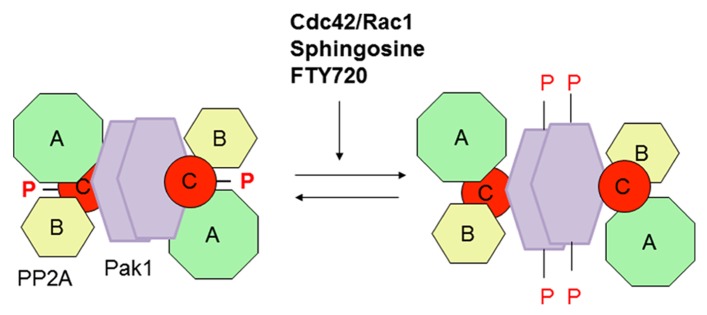
**Activation of phosphatase PP2A by Pak1 scaffolding.** Through a conformational change associated with Pak1 autophosphorylation, PP2A undergoes auto-dephosphorylation with increased phosphatase activities. Pak1 remains in dimer form and associated with PP2A even after auto-phoshporylation.

The constitutively active Pak1 variant co-purifies with PP2A from mammalian cell lines, but it does not phosphorylate the PP2A in vitro (Ke et al., 2004). In addition, constitutively active Pak1 does not phosphorylate PP2A isolated from a muscle source *in vitro*. These results indicate that activation of PP2A may be through a scaffolding mechanism (Ke et al., 2008; **Figure 3**). PP2A dephosphorylates multiple targets and is involved in regulation of cardiac excitation and contraction. Moreover in Pak1 knock-out mice, phosphorylation of cofilin at serine-3 was elevated with altered regulation of glucose homeostasis (Wang et al., 2011b).

## ANTI-ADRENERGIC ACTIVITY OF Pak1 IN THE HEART

In cardiomyocytes, PKA phosphorylation is often reversed by PP2A. Activation of Pak1/PP2A thus represents a novel molecular mechanism that antagonizes β-adrenergic signaling in the heart. Rhythmic and effective contraction and relaxation of myocardium requires integration of electrical signals, Ca^2+^ transients and myofilament cross-bridge cycling, which are all subjected to regulation of protein kinases and phosphatases (Lei et al., 2007; Sheehan et al., 2007; Ke et al., 2008). β-adrenergic signaling pathways mediated by cAMP dependent protein kinase induce increased phosphorylation of L-type Ca^2+^ channels and ryanodine receptors leading to enhanced Ca^2+^ inflow and Ca^2+^ induced Ca^2+^ release from sarcoplasmic reticulum (Kamp and Hell, 2000; Ke et al., 2008); phosphorylation of troponin I at serine 23, 24 accompanied by accelerated cross-bridge detachment and myocardial relaxation (Solaro et al., 2013); phosphorylation of phospholamban, which renders faster SERCA2a Ca^2+^ reuptake activity. PP2A usually removes phosphate groups from PKA targets at the same phosphorylation sites (Ke et al., 2008). In cultured adult rat ventricular myocytes, expression of constitutively active Pak1 induces dephosphorylation of cardiac troponin I, myosin binding protein C and increased Ca^2+^ sensitivity of myofilament force development. In SA nodal cells, Pak1 inhibits isoproterenol (ISO) induced increases of pacemaker activity through regulation of activities of L-type Ca^2+^ and delayed rectifying potassium channels (Ke et al., 2007a). In mouse with complete ablation or cardiac specific knock-out of Pak1, there is an accelerated progression to hypertrophy in response to stresses induced by chronic treatment with ISO or by transverse aortic constriction (TAC; Liu et al., 2011; Taglieri et al., 2011).

There is also evidence that active Pak1 acts as antagonist of angiotensin II signaling. In neonatal cardiomyocytes, mechanical or hormone-induced hypertrophy can be blocked by type I angiotensin II receptor antagonists (Yamazaki et al., 1996), and checked by Rho inhibitors (Hoshijima et al., 1998) and inhibited by expression of constitutively active Pak1 (Liu et al., 2011). Pak1 may also have a beneficial role in improving cardiac functions during endotoxemia by blocking a TNF signaling pathway involving activation of another protein phosphatase mitogen-activated protein kinase phosphatase-1 (MKP-1; Zhang et al., 2012). On the other hand, Pak1 may contribute to expression of inflammatory markers on endothelium at atherosclerosis-susceptible regions of arteries *in vivo* (Jhaveri et al., 2012). To confirm and extend all the above observations in larger animals and in humans, development of potent and specific molecular tools targeting Pak1 activation will be important and valuable.

## IMPLICATION IN THERAPEUTICS AND DRUG DEVELOPMENT

Knowledge related to the extensive molecular interactions between the Pak1 monomers and the Pak1 kinase domain, which has the capacity to interact with and phosphorylate multiple sequences in the N-terminal half of the kinase, has provided great potential to design peptides targeting Pak1 *in vitro* and *in vivo.* The peptide sequences surrounding or close to auto-phosphorylation sites are especially interesting inasmuch as all the auto-phosphorylation sites along with the adjacent amino acids interact with the Pak1 kinase domain. Based on crystal structures, most of the interaction of the N-terminal motifs with the Pak1 kinase domain occurs in “trans” or through an inter-molecular mechanism between two Pak1 monomers. A Pak1 interacting peptide has to compete with the cognate or native peptide (the corresponding peptide sequence inside the Pak1 molecule) for the same binding region in Pak1. This competition could dislocate the “native peptides” and attenuate the Pak1–Pak1 interaction. Thus, interruption of Pak1 homodimer and attenuation of auto-inhibition may occur. PRR-1 and PID are bioactive peptides interacting with Pak1 (Zenke et al., 1999; Ke and Solaro, 2008), and both containing auto-phosphorylatable amino acids. PRR-1 is derived from the Pak1 first proline-rich repeat that promotes Pak1 translocation from the plasma membrane to cytosolic fractions (Kiosses et al., 2002). PRR-1 reduces phosphorylation of myosin regulatory light chain in endothelial cells (Stockton et al., 2004). The same effect was produced by constitutively active Pak1 in the same cell type (Ke et al., 2007b). PID is derived from Pak1 inhibitory domain, which lies immediately downstream and even overlaps with PBD. Although it was regarded as a Pak1 inhibitor, the peptide actually reduced paxillin density at the cell periphery (Delorme-Walker et al., 2011). The same phenotype was observed in mammalian cells expressing constitutively active Pak1 (Manser et al., 1997), Pak18 is a peptide derived from the region that interacts with Pix. The Pix interacting region must also be able to interact with the Pak1 kinase domain because it contains two auto-phosphorylation sites. Interestingly, the peptide reduced phosphorylation of MLC_20_ (Santiago-Medina et al., 2013), a functional effects produced by expression of constitutively active Pak1 (Sanders et al., 1999; Ke et al., 2007b).

Studies of Pak1 mutants indicate that mutations of Pak1 often lead to gain of Pak1 activities. For example, mutation of amino acids in Pak1 dimerization region H81 and H86 attenuate Pak1 dimerization and increases auto-phosphorylation and Pak1 activity on cytoskeletal reorganization. Change of an amino acid in PBD domain, L107F has an even stronger effect in activating Pak1. Similarly, switch of serine and threonine at auto-phosphorylation sites to acidic amino acids mimicking phosphorylation increases Pak1 activity. Truncation of amino acids at the Pak1 N-terminal region (Frost et al., 1998) and cleavage of Pak2 increases auto-phosphorylation (Walter et al., 1998) and provide a means of gain of Pak1 activities. In frog embryo, microinjection of protease activated Pak2 significantly inhibited cell division (Rooney et al., 1996). Pak1 and Pak3 are highly homologous to each other. Mutations of amino acids R67C and A365E in Pak3 located distantly from each other result in the same genetic defects, mental retardation in humans (Bienvenu et al., 2000; Gedeon et al., 2003). Human Pak3 MRX30 mutation with premature termination of Pak3 translation induced an increase of filopodia-like protrusions and long spines in pyramidal neurons (Boda et al., 2004). Whether these mutations could be traced to the same enzymatic activity change or change in signaling activity requires further investigation.

**FIGURE 4:**
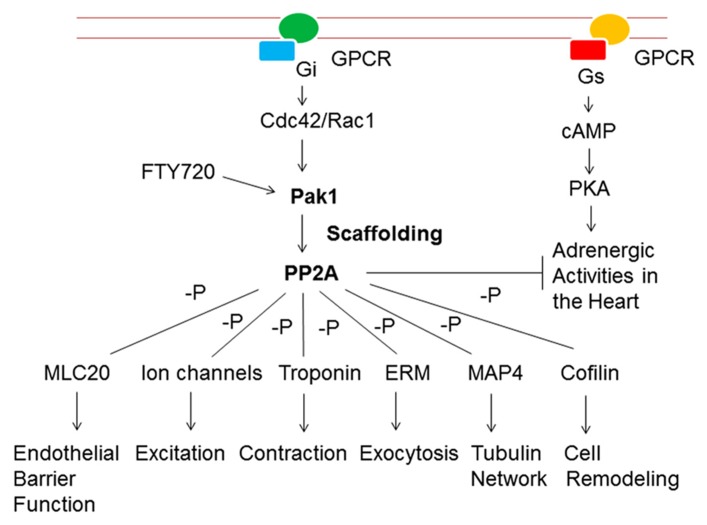
**Scaffolding of Pak1-PP2A induces different functional changes in endothelial, cardiac and other mammalian cells.** It is also a novel anti-adrenergic mechanism in the heart. The Pak1-PP2A scaffolding can be enhanced by small molecular weight compounds such as FTY720. See text for further discussion.

As a key regulatory molecule crucial for multiple physiological processes, it is not surprising that Pak1 is regulated at multiple levels by diverse mechanisms (**Figure 4**). Activation of Pak1 by low molecular weight molecules exists in nature, and it is likely that some of those may not have been discovered yet. Pak1 is directly activated by sphingosine* in vitro* and *in vivo* characterized by an increase of auto-phosphorylation, bypassing upstream protein signals (Gedeon et al., 2003; Jhaveri et al., 2012). Sphingosine is enriched in lipid rafts and exists in all mammalian cells regulating diverse cellular processes including pacemaker activities when converted to sphingosine phosphate (Boda et al., 2004; Strader et al., 2011). C2 and C6 ceramides are PP2A activators and structurally similar to sphingosine. They both activate Pak1 *in vitro *(Ke and Solaro, 2008). A sphingosine analog, FTY720, was derived from myriocin (ISP-1), a metabolite from fungus *Isaria sinclairii*, a Chinese traditional medicine with a variety of therapeutic applications (Adachi and Chiba, 2008; Strader et al., 2011). In humans, FTY720 induces bradycardia and antagonizes the chronotropy of ISO (Kovarik et al., 2008a,b). In *ex vivo* rat heart, FTY720 normalizes arrhythmias triggered by enhanced β-adrenergic stimulation through Pak1 activation (Brinkmann et al., 2002). A recent study also indicates that FTY720 effectively prevents dendrite loss in a mouse model of experimental autoimmune encephalomyelitis (Rossi et al., 2012).

FTY720 is converted to FTY720-phosphate by sphingosine kinase and then binds to sphingosine-1 phosphate receptors (Brinkmann et al., 2002), which has been considered as the major signaling pathways *in vivo*. However, in endothelial cells, inhibition of sphingosine-1 phosphate receptors did not abolish FTY720 induced enhancement of barrier integrity (Dudek et al., 2007), suggesting FTY720 protects endothelial barrier function through a different pathway. A similar observation was made in the heart that FTY720 protects an ischemic heart by post-conditioning similar to sphingosine, but independent of either sphingosine kinase or S1P receptors (Vessey et al., 2013). In *ex vivo* hearts, FTY720 treatment induces an increase in Pak1 auto-phosphorylation (Egom et al., 2010). The *ex vivo* heart responds to FTY720 instantly, which may suggest that Pak1 is directly activated by FTY720 (Egom et al., 2010). Identification of a more specific agonist that turns on Pak1 activities *in vivo* will not only have application to further define Pak1 function *in vivo*, but also may serve as a novel therapeutic approach for cardiovascular diseases. FTY720 activates PP2A and inhibits tumor cell growth and tumorigenesis (Liu et al., 2008; Yang et al., 2012; Saddoughi et al., 2013). In mice TAC induced cardiac hypertrophy can be both inhibited and reversed by FTY720. The anti-hypertrophic effects are abolished in mouse models with cardiac specific knock-out of Pak1 (Liu et al., 2011). Although some efforts have been made to understand the molecular mechanism whereby Pak1 is turned on by sphingosine (Zenke et al., 1999; Chong et al., 2001), activation of Pak1 by compounds is an under-explored field. Because an autoinhibitory configuration of Pak1 is maintained through multiple sequences throughout the whole molecule, it will not be surprising that Pak1 activation or release of Pak1 auto-inhibition can be realized by molecules that are structurally very different.

## CONCLUSION

Employment of scaffolding in signal transduction by Pak1 challenges the conventional understanding of a protein kinase. Some protein kinases other than Pak family members may also use the same signaling mechanism, but are not yet uncovered. Better understanding of scaffolding of Pak1 not only provides novel insights and enriches our knowledge on diverse modes of signal transduction, but also serves as guide for drug design and therapeutics targeting at Pak1. Substantial efforts have been made to develop inhibitors for Paks. Highly selective agonists for Pak1 may be equally important. The unique structure and catalytic activity of Pak1 has provided great potential for design and development of Pak1 activators through disruption of Pak1 auto-inhibition. These molecular tools should be valuable in further exploring Pak1 function *in vivo* and serve as drug candidates for novel therapeutics of major cardiovascular diseases.

## Conflict of Interest Statement

The authors declare that the research was conducted in the absence of any commercial or financial relationships that could be construed as a potential conflict of interest.
